# Robotic-Assisted Minimally Invasive Resection of Multiple Oesophageal Schwannomas: A Case Report

**DOI:** 10.7759/cureus.74589

**Published:** 2024-11-27

**Authors:** Jack Menzie, Thomas Eldredge, Liang Low

**Affiliations:** 1 Department of General Surgery, Monash Health, Melbourne, AUS; 2 Department of Upper Gastrointestinal and Hepatobiliary Surgery, Monash Health, Melbourne, AUS

**Keywords:** esophageal tumor, gastro-esophageal surgery, minimally invasive surgical procedures, robotic surgical management, robotic surgical procedures, robotic thorascopic surgery, schwannoma, surgical enucleation, upper gi surgery, upper gi tumor

## Abstract

Schwannomas are rare, benign tumours arising from Schwann cells, with oesophageal cases representing a small fraction. Their variety of symptoms and nonspecific imaging features often make preoperative diagnosis challenging, frequently requiring immunohistochemical staining for confirmation. We describe the case of a 62-year-old woman with progressive dysphagia, found to have a subepithelial mass at the gastroesophageal junction (GOJ). Imaging and endoscopic ultrasound-guided biopsy confirmed an oesophageal schwannoma. The patient underwent robotic-assisted thoracoscopic excision, which allowed for precise tumour removal while avoiding a more extensive oesophagectomy. Intraoperatively, two distinct lobular tumours were identified and successfully excised without compromising the oesophageal mucosa. Postoperatively, the patient recovered well with resolution of symptoms and no evidence of residual tumour on follow-up imaging. This case highlights the potential of robotic-assisted approaches for treating oesophageal schwannomas, which can offer advantages in complex resections by improving surgical precision and reducing morbidity. Despite promising outcomes, robotic-assisted enucleation of oesophageal schwannomas remains rare, with few cases documented. This case supports robotic resection of oesophageal schwannomas as a feasible option in specialized settings, but further studies are needed to establish its role and develop recommendations.

## Introduction

Schwannomas are slow-growing, mostly benign tumours arising from neuronal Schwann cells [1-3]. Occurrence in the gastrointestinal (GI) tract is considered rare, representing <1% of all tumours of the GI tract and 2-6% of all GI mesenchymal tumours [1,2,4]. They are most commonly located in the stomach and colorectum, with few reports of oesophageal schwannomas [1,5].

Oesophageal schwannomas may be incidentally identified but can also cause a variety of symptoms, including cough, dysphagia, chest/abdominal pain, dyspnoea, bleeding or unexplained weight loss [4]. Progressive dysphagia, due to the indolent and slow-growing nature of the tumour, and dyspnoea in the presence of tracheal compression are the most commonly reported symptoms [4,6]. Female patients aged over 50 years demonstrate a preponderance for developing oesophageal schwannomas, with a possible increased prevalence in Asian populations [4,7].

Schwannomas are difficult to differentiate from other mesenchymal tumours, such as leiomyomata or gastrointestinal stromal tumours (GIST), with definitive identification often not established until after resection [4]. The featureless and non-specific imaging characteristics of schwannomas make radiological diagnosis challenging [8]. 18-Fluorodeoxyglucose positron emission tomography (FDG-PET) may aid in detection, as nerve cells express glucose transporter type 3 and FDG uptake is increased from these transporters giving a hypermetabolic appearance, but this does not provide a diagnosis [4,9]. In addition, endoscopic ultrasound-guided fine-needle aspiration (EUS-FNA) has been shown to aid in the characterisation of schwannomas and other submucosal lesions of the upper GI tract but has limited accuracy [10]. Therefore, to get a diagnosis of schwannoma immunohistochemical staining is required; positivity for S100 protein or Sox10 supports the possibility of nerve sheath origins, but staining needs to be negative for actin, desmin, caldesmon, DOG-1 and CD117, which support smooth muscle origins [2].

Due to the rarity of the condition, lack of comprehensive studies and deficiency of diagnostic criteria, management decisions are left to a clinician’s discretion, often with progression to surgical resection in cases where the tumour is large, increasing in size, or symptomatic, or there are concerns for malignant progression [11].

The presented case outlines a patient with symptomatic, biopsy-proven, oesophageal schwannomas managed with robotic-assisted minimally invasive resection with excellent recovery and resultant symptom resolution.

## Case presentation

**Figure 1:**
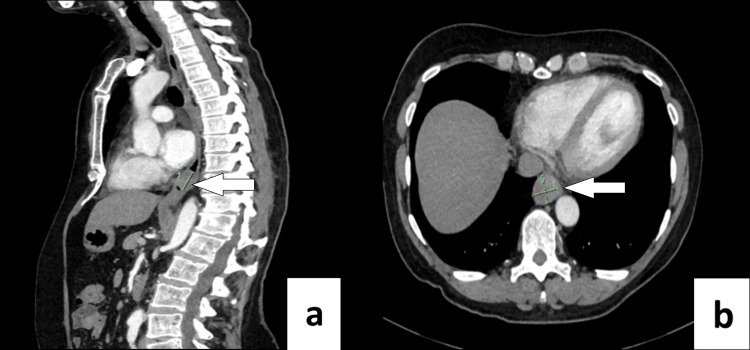
Pre-operative Computed Tomography (CT) scan Pre-operative Computed Tomography (CT) scan (a. sagittal, b. axial views) of a distal oesophageal mass likely representing a schwannoma, measuring approximately 2.2 cm x 2.4 cm transaxially and approximately 2.4 cm in craniocaudal length. White arrows indicate distal oesophageal mass.

A 62-year-old Caucasian female with no significant past medical history or relevant family history was referred with a history of progressive dysphagia spanning several years. Prior computed tomography (CT) images from 2018 demonstrated a mass in the distal oesophagus, with follow-up CT images in 2022 (Figure 1) showing interval enlargement and favoured to represent a sub-mucosal tumour.

**Figure 2:**
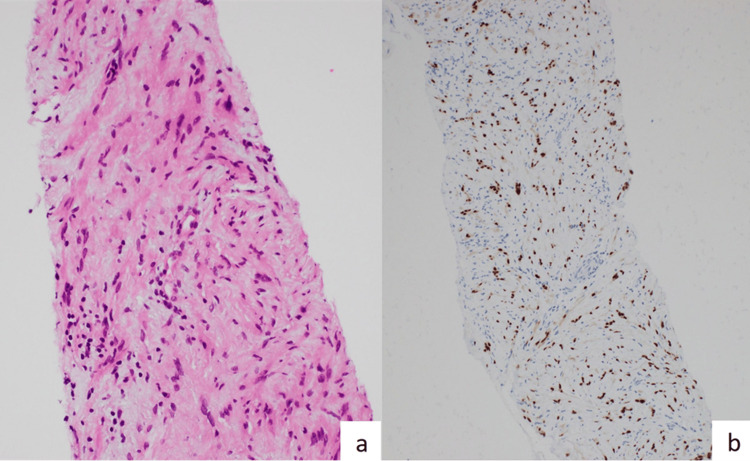
Cytopathology Cytopathology (a. Hematoxylin and eosin stain, b. SOX-10 antibody immunohistochemistry) showing scattered fragments of spindled cells positive for SOX10, consistent with a schwannoma.

Gastroscopy revealed a subepithelial mass at the gastroesophageal junction (GOJ) and mucosal biopsies were normal. Subsequently, an endoscopic ultrasound (EUS) was performed using a linear echoendoscope, identifying a hypoechoic 2.5 cm subepithelial lesion at the gastroesophageal junction (GOJ). Fine needle aspiration was undertaken with a 22 gauge Acquire™ needle (Boston Scientific, Massachusetts, USA). Two passes were taken and sent for cytology and immunohistochemistry. CD34, C-kit, CAM5.2, desmin, and progesterone receptor were negative, however, cytopathology showed scattered fragments of spindled cells positive for SOX10, consistent with a schwannoma (Figure 2).

The patient proceeded with a robotic-assisted minimally invasive thorascopic local excision of the tumour to avoid a sub-total oesophagectomy. Four robotic ports and an assistant port were used, the right lung was isolated, and the robot docked. The right inferior pulmonary ligament was divided and the tumour was identified. The parietal pleura was then divided over the oesophagus, and a longitudinal oesophageal myotomy was performed over the tumour, at which time two distinct lobular tumours were identified. Both tumours were excised intact, taking care not to breach the oesophageal mucosa. The myotomy was then closed using a continuous 3/0 Stratafix suture (Johnson & Johnson MedTech, Warsaw, USA). Haemostasis was ensured and 24-French intercostal drain was inserted.

**Figure 3:**
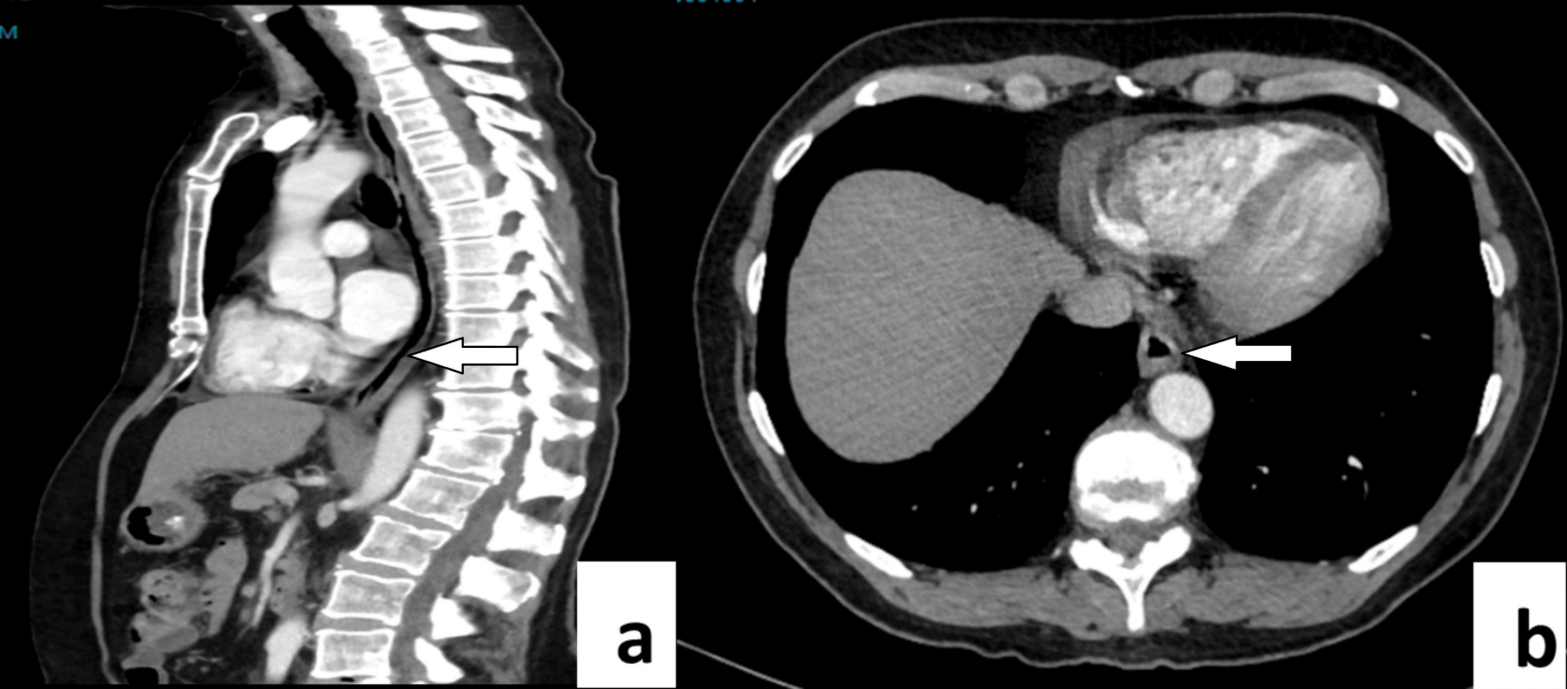
Post-operative Computed Tomography (CT) scan Post-operative Computed Tomography (CT) scan (a. sagittal, b. axial views) 12 months post robotic-assisted minimally invasive resection of multiple oesophageal schwannomas. White arrows indicate the site of the previous distal oesophageal mass.

Post-operatively, the patient was managed in the surgical ward and had an uncomplicated recovery. A contrast swallow test was performed revealing no leak or additional post-operative complications. Once tolerating oral diet, she was discharged home after four post-operative days. A clinical review was undertaken 1-month and 4-months post-operatively, with the patient feeling entirely well with complete resolution of her pre-operative symptoms. A surveillance CT scan and review were arranged at 12 months’ time to assess progress post-resection. The CT (Figure 3) revealed no evidence of residual oesophageal mass, lymphadenopathy, or evidence of metastatic disease in the chest, abdomen, or pelvis. The patient continued to remain free of her pre-operative symptoms and reported improved quality of life.

## Discussion

The rarity of oesophageal schwannomas is reflected by a paucity of reports in the medical literature [1,2,12]. As such, specific management guidelines or diagnostic criteria are lacking, and in most cases diagnosis is only made after resection [4]. Additionally, patients presenting with symptoms such as cough, dysphagia, chest pain, abdominal pain, dyspnoea, bleeding, weight loss are relatively non-specific [4,7]. Clinical imaging is also non-specific due to schwannomas having featureless characteristics making delineation from other tumours or even identification of a tumour difficult [8]. When identification of an oesophageal schwannoma or sub-mucosal tumour is made, the decision is often left to the surgeon to decide management, with surgical resection often being considered the main treatment for oesophageal schwannomas [12-14].

In the current literature, decisions on the therapeutic management of oesophageal tumours depend on several factors including symptoms the patients are experiencing, tumour size, rate of growth and its potential to develop malignancy [14,15]. Based on these factors most studies advocate for surgical resection as treatment [8,15,16]. Schwannomas in particular have been found to be managed with enucleation, esophagectomy and in some cases via an endoscopic approach [15-17]. The main approach for surgical removal of oesophageal schwannomas is video-assisted thoracoscopic surgery (VATs) followed by posterolateral thoracotomy and then robotic-assisted thoracoscopic surgery [14,15]. Souza et al. found that enucleation is the most common procedure (59.2%), followed by subtotal esophagectomy (7.4%) and endoscopic removal (5.5%) [15]. While endoscopic approaches have been used, they are often only effective for small (<2cm), well-circumscribed lesions in the submucosal tissue [16]. Management of large lesions (2-8 cm) will generally be a surgical approach via VATs/thoracotomy for mid to distal regions of the oesophagus [16]. When encountering lesions greater than 8 cm the literature recommends oesophagectomy as the surgical approach [16].

Given that enucleation is the most common technique there are number of case studies that describe its outcomes. However, given that schwannomas are the rarest of sub-mucosal tumours there are very few case studies in the literature. Additionally, there are fewer still that describe schwannomas that have been removed at the distal oesophagus via robotic assistance [12,14].

The first robotic case for enucleation was described by Elli et al. in 2004. Since then there have been two case series describing robotic-assisted enucleation of submucosal tumours, and of the cases in both series, only one involved a schwannoma of the distal oesophagus [12,14,18]. The singular case by Zhang et al. is the only case study we found that involves robotic-assisted enucleation of a schwannoma, and similarly to our case, involves the distal oesophagus [12]. In this case, a robotic-assisted enucleation of a large (7 cm) dumbbell-shaped schwannoma was successfully performed with an operative time of 108 minutes - markedly less when compared to other enucleation techniques such as VATs/thoracotomy, which, from the limited studies available, range from 138 - 498 minutes [12,19,20]. Comparatively, our case involved not a single schwannoma as originally thought but two in the submucosal plane of the distal oesophagus, thereby increasing the difficulty and risk of mucosal injury at the time of enucleation. Similarly, a case series by Froiio et al. performed six robotic-assisted cases on submucosal tumours, four of which were enucleations [14]. One of the enucleated cases was performed on a singular 6.5 x 4.7 cm tumour initially thought to be a GIST involving the proximal oesophagus. This particular case is mentioned as it was later found post-operation to be a schwannoma on histopathology. What is interesting about this case is that it adds to the evidence that large tumours may be enucleated safely and may avoid the need for oesophagectomy. Oesophagectomy has previously been recommended for tumours >5-8cm [11,16].

Despite the rarity of cases, enucleation has been described in the literature as the accepted approach for benign tumours of less than 5 cm [14,21,22]. Whereas larger tumours or tumours that provide technical challenges for the surgeon have been assigned to oesophagectomy [14,21,22]. The introduction of thoracoscopic approaches has improved the ability of surgeons to enucleate tumours but, as described by Watanabe et al., larger tumours (>5cms) and their location still provide challenges to successful enucleation without damage to the oesophageal wall and subsequent conversion to oesophagectomy [11]. Large (7 cm), irregular-shaped tumours may still be amenable to enucleation with robotic assistance without the need to convert to oesophagectomy as seen by Zhang et al. [12,14]. Additionally, our case presented here provides support that when encountering unforeseen intraoperative findings, such as multiple tumours, which increase the difficulty of complete enucleation without damage to the oesophageal wall, robotic-assisted approaches may be a viable option.

The robotic-assisted approach provides advantages over conventional laparoscopic and thoracoscopic techniques such as 3-dimensional viewing, improved articulation of surgical instruments in confined spaces, and improved dissection and suturing technique. Smooth wrist movements may aid in preventing oesophageal injuries that can be incurred from a surgeon’s hands. Moreover, in cases of robotic-assisted enucleations patients have had shorter operative times, reduced length of stay in the hospital and few if any intra/post-operative complications [12,14]. Whilst these results are promising, there are only two cases of schwannomas being enucleated by robotic assistance in the literature and a small number of cases looking at other submucosal tumours, thus, there is little established evidence for physicians to compare management pathways and advocate for this approach currently [12,14]. 

While robotic-assisted enucleation of submucosal tumours is emerging and shows promise, there are notable limitations in the current literature. Guidance on this approach is sparse, and the few existing studies differ in the size and type of tumours suitable for enucleation versus resection. Furthermore, robotic procedures are typically performed only in specialized centres, restricting both patient access and the availability of trained surgeons. The literature also lacks consensus on definitions for large or complex tumours, complicating meaningful comparisons between cases.

## Conclusions

Oesophageal schwannomas are rare submucosal tumours with varied symptoms and potential malignancy, often challenging to diagnose due to limitations in current imaging and management pathways. Our case suggests that once a tumour is identified, careful planning in a specialist centre by a trained surgeon may provide a diagnosis and a minimally invasive approach to treat oesophageal tumours. We suggest that by the use of a robotic-assisted approach, unforeseen complications for larger or multiple tumours can still be safely managed. To support this conclusion, further cases are needed that include a larger number of participants, with varying sizes of schwannomas and their locations in the oesophagus, to compare before any appropriate recommendations can be advised.
